# Supplementary material for: An Application of Machine Learning That Uses the Magnetic Resonance Imaging Metric, Mean Apparent Diffusion Coefficient, to Differentiate between the Histological Types of Ovarian Cancer
Source: J Clin Med. 2021 Dec 31;11(1):229. doi: 10.3390/jcm11010229 (PMC8745699; doi:10.3390/jcm11010229)
Supplement: Supplementary file 1 [file jcm-11-00229-s001.zip › jcm-1459556-supplementary.pdf]

Supplementary Materials

Table S1. Characteristics of study population for stage Ⅰ EOC including BOT (*n* = 82).

	**HGSOC (*n* = 10)**	**Non-HGSOC (*n* = 72)**	***p* value**
Age at diagnosis (years)	52 [50, 60] [1]	49 [41, 57] [1]	0.036
Overall survival (months)	83	43	0.71
Histologic types			
HGSOC	10 (100%)	0	
EC	0	11 (15.3%)	
CCC	0	25 (34.7%)	
LGSOC+BOT	0	9 (12.5%)	
MC	0	27 (37.5%)	
CA 125 (IU/ml) [2]	66.1 [22.4, 134] [1]	31.5 [12.2, 94.6] [1]	0.094
≤ 35	3 (30%)	31 (41.7%)	
> 35	6 (60%)	29 (40.3%)	
CA 19-9 (IU/ml) [2]	7.41 [3.62, 17.2] [1]	29.0 [9.7 71.5] [1]	0.015
≤ 37	8 (80%)	29 (40.3%)	
> 37	0	21 (29.2%)	
ADC_{mean} ($\times 10^{-3} s/m^2$)	1.13 [1.01, 1.70] [1]	1.56 [1.32, 1.79] [1]	0.007
Total diameter (cm)	9.2 [6.6, 15.8] [1]	11.0 [7.3, 13.1] [1]	0.83
Ratio (solid/total cyst, area)	0.11 [0.023, 0.39] [1]	0.17 [0.052, 0.48] [1]	0.22
Location of ovarian lesion			0.21
Both	0		
Right	4 (40%)	44 (61%)	
Left	6 (60%)	28 (39%)	
Peritoneal seeding			0.48
Present	0	7 (9.7%)	
Absent	10 (100%)	65 (90.3%)	

[1] Median [25percentile, 75percentile]. HGSOC: High grade serous ovarian cancer, ADC_{mean}: mean value of apparent diffusion coefficient, EOC: Epithelial ovarian cancer HGSOC: High-grade serous ovarian cancer, EC: Endometrioid cancer, CCC: Clear cell carcinoma, LGSOC: Low-grade serous ovarian cancer, MC: Mucinous carcinoma, BOT: Borderline ovarian tumor.

Figure S1. ADC_{mean} and the ratios of the area of the solid portion to the total area of ovarian. Lesions according to histological types of stage Ⅰ EOC including BOT (n = 82).

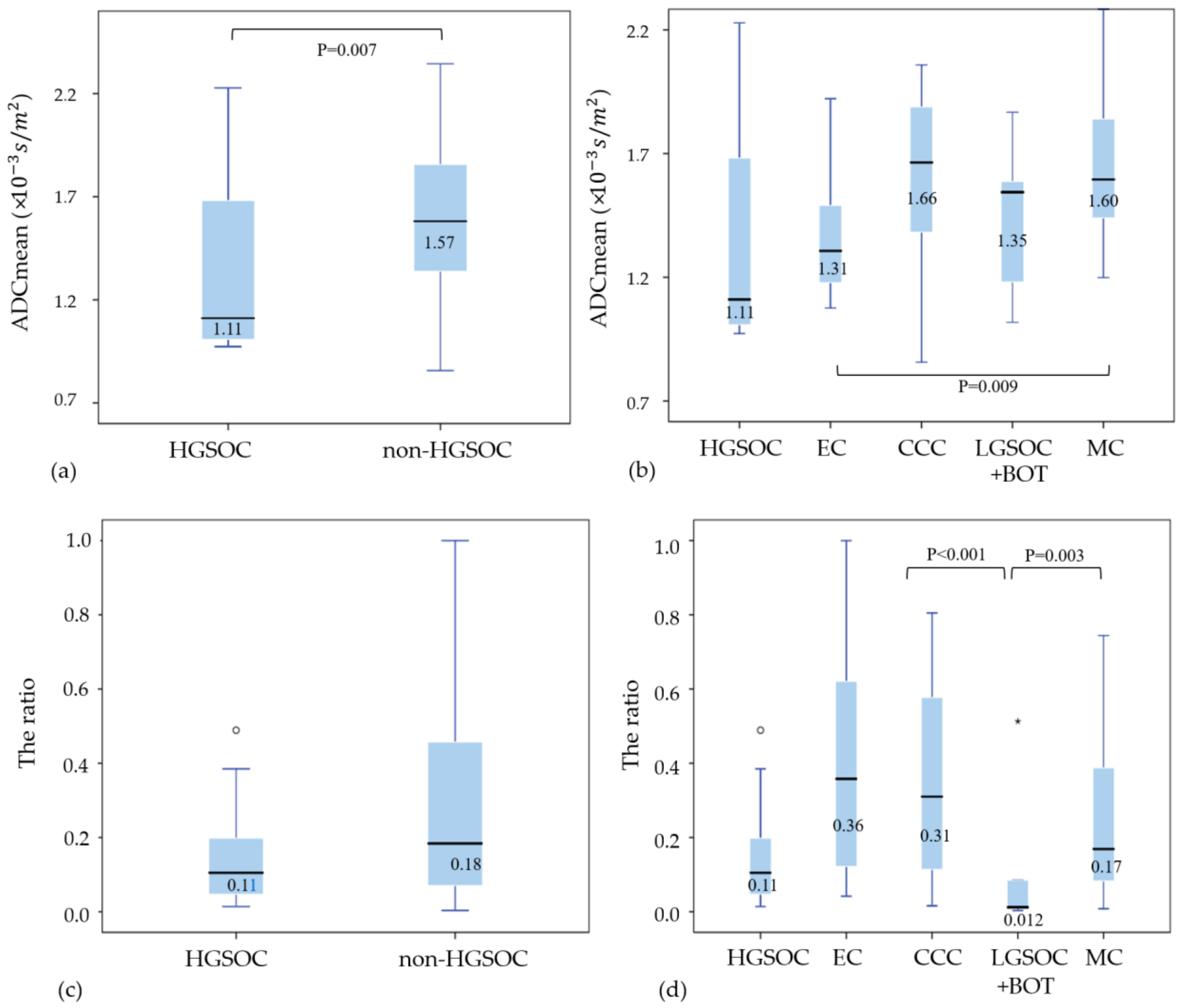

(**a**) Between HGSOC and non-HGSOC, (**b**) Among 5-histologic types of EOC (**c**) The ratio of area of solid portion to total ovarian lesion between HGSOC and non-HGSOC, (**d**) The ratio of area of solid portion to total ovarian lesion among 5-histological types of EOC. EOC: Epithelial ovarian cancer, HGSOC: High-grade serous ovarian cancer, EC: Endometrioid cancer, CCC: Clear cell carcinoma, LGSOC: Low-grade serous ovarian cancer, MC: Mucinous carcinoma, BOT: Borderline ovarian tumor
